# Supplementary material for: Novel Role of CETP in Macrophages: Reduction of Mitochondrial Oxidants Production and Modulation of Cell Immune-Metabolic Profile
Source: Antioxidants (Basel). 2022 Aug 31;11(9):1734. doi: 10.3390/antiox11091734 (PMC9495589; doi:10.3390/antiox11091734)
Supplement: Supplementary file 1 [file antioxidants-11-01734-s001.zip › antioxidants-1896304-supplementary.pdf]

Supplementary Material

Novel Role of CETP in Macrophages: Reduction of Mitochondrial Oxidants Production and Modulation of Cell Immune-Metabolic Profile

Gabriel G. Dorighello, Leandro H. P. Assis, Thiago Rentz, Joseane Morari, Monique F. M. Santana, Marisa Passarelli, Neale D. Ridgway, Anibal E. Vercesi and Helena C. F. Oliveira

A: CETP genotyping: tail tip genomic DNA

B: CETP mRNA

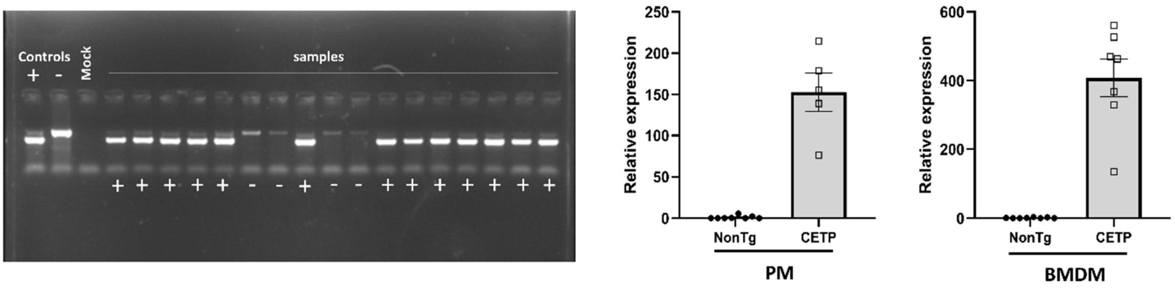

Supplementary Figure S1: CETP genotyping (A) by PCR of genomic DNA and mRNA expression (B) in peritoneal macrophage (PM) and bone marrow derived macrophage (BMDM).

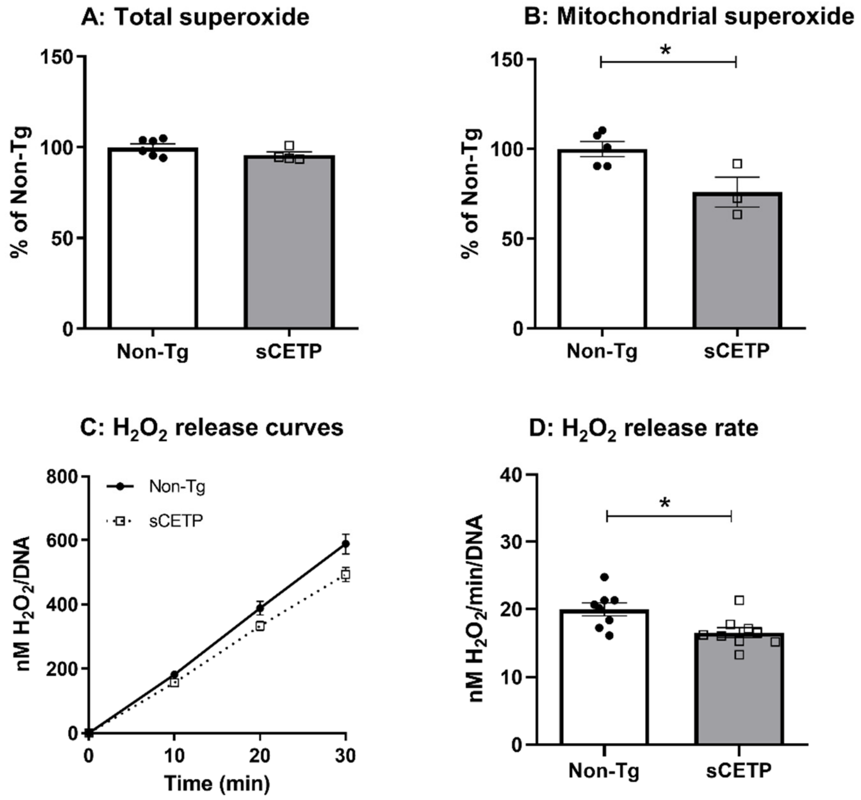

Supplementary Figure S2: Oxidant production in thioglycolate-elicited peritoneal macrophages from simian CETP transgenic mice and non-transgenic mice (Non-Tg). Total cell superoxide quantified with DHE (**A**), mitochondrial superoxide quantified with Mitosox® (**B**), total hydrogen peroxide release rates (**C-D**) quantified with Amplex® red and HRP. A: n=4-6 for cell superoxide; B: n=3-5 for mitochondrial superoxide; C-D: n=8-9 for H_2O_2 release; *p<0.05 vs. Non-Tg.

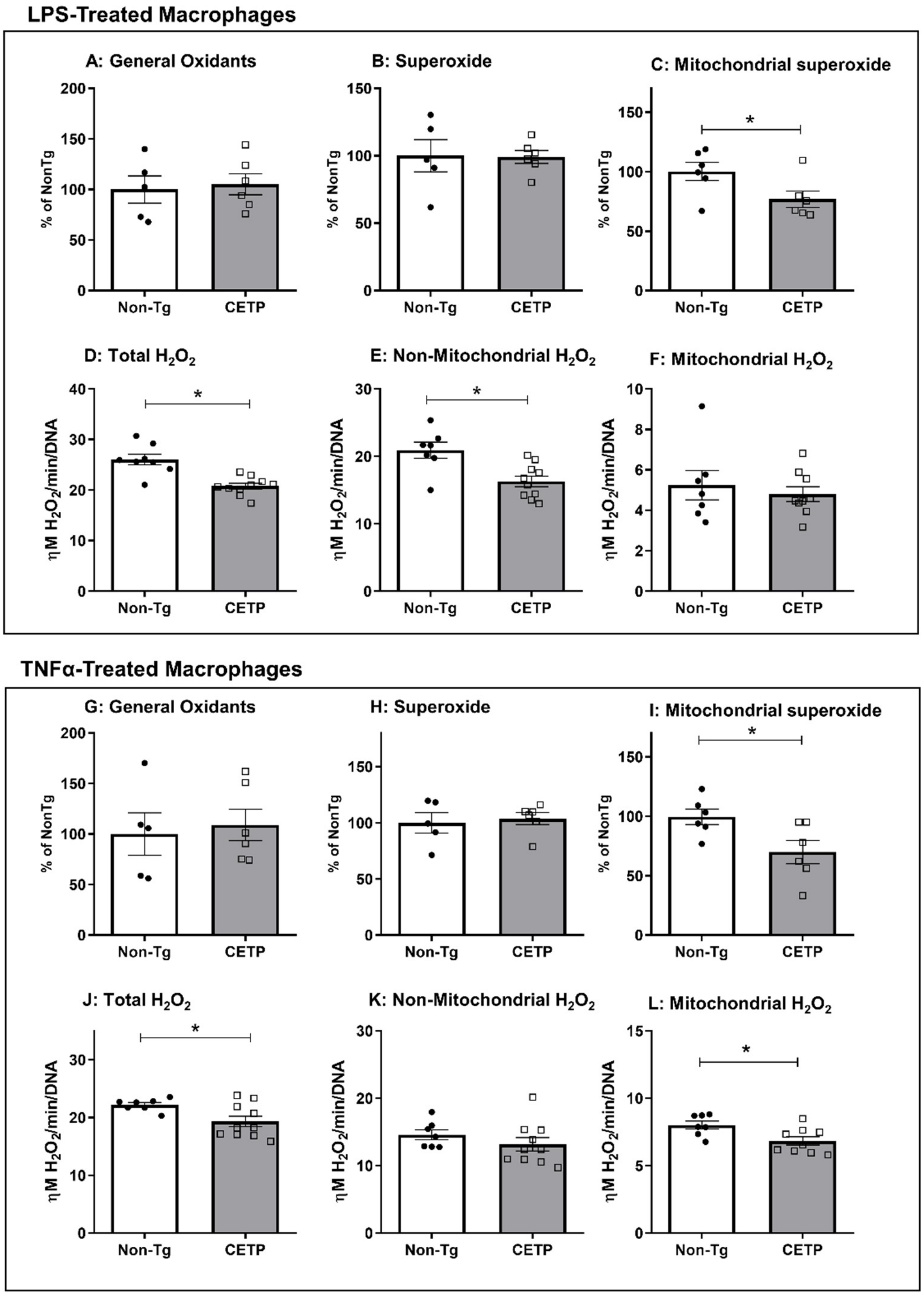

Supplementary Figure S3: Oxidant generation in thioglycolate-elicited peritoneal macrophages from human CETP transgenic mice and non-transgenic mice (Non-Tg) after activation with LPS (100 nM) (**A-F**) or TNFα (5 U/mL) (**G-L**). General oxidants quantified with H_2DCFDA (**A**, **G**), total cell superoxide quantified with DHE (**B**, **H**), mitochondrial superoxide quantified with Mitosox® (**C, I**), total H_2O_2 (**D, J**), non-mitochondrial H_2O_2 (**E, K**) and mitochondrial H_2O_2 (**F, L**) with Amplex® red and HRP. Non-mitochondria H_2O_2 contribution was determined in cells treated with FCCP (1 µM) and mitochondrial derived H_2O_2 was calculated as [total - non-mitochondrial H_2O_2]. N=5-6 for global oxidants and superoxide; n=7-10 for H_2O_2 release; *p<0.05 vs. Non-Tg.

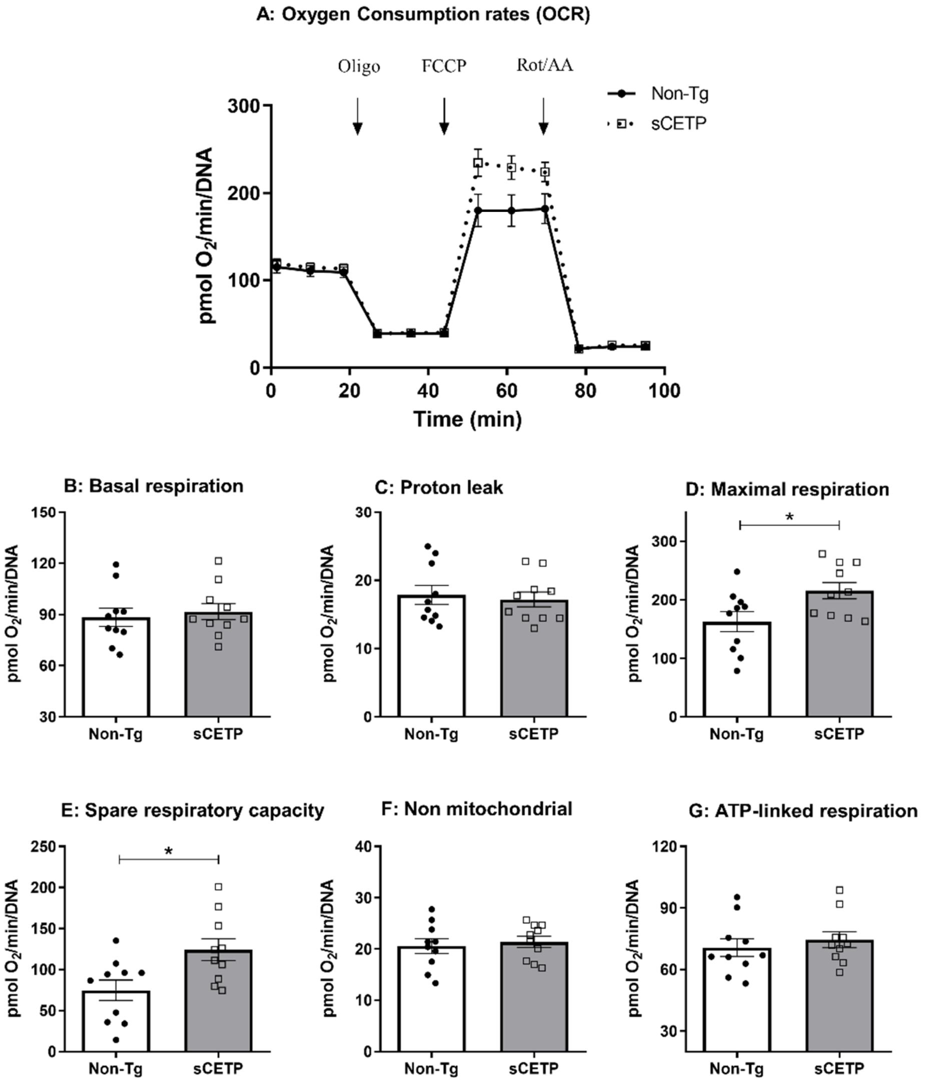

Supplementary Figure S4: Oxygen consumption rates (OCR) in thioglycolate-elicited peritoneal macrophages from simian CETP (sCETP) transgenic and non-transgenic mice (Non-Tg). The OCR were evaluated using a Seahorse® - XF24 Extracellular Flux Analyzer (Agilent, USA). **A**: Average curves of OCR with sequential additions of oligomycin (1 µM), FCCP (1 µM), and rotenone/antimycin A (1 µM); **B**: Basal respiration; **C**: Proton leak; **D**: Maximal respiration; **E**: Spare respiratory capacity; **F**: Non-mitochondrial respiration; **G**: ATP linked respiration; n= 10; *p<0.05 vs. Non-Tg.

Supplementary Table S1. Oligonucleotides used to assess gene expression.

Gene	Oligonucleotides sequences	
ACTB	forward	5’-AGAAGCTGTGCTATGTTGCTCTA-3’
	reverse	5’-TCAGGCAGCTCATAGCTCTTC-3’
hCETP	forward	5’-GGCCAAGTCAAGTATGGGTTG-3’
	reverse	5’-ACAGACACGTTCTGAATGGAGA-3’
ARG1	forward	5’-CTCCAAGCCAAAGTCCTTAGAG-3’
	reverse	5’-AGGAGCTGTCATTAGGGACATC-3’
CD36	forward	5’-GGAACTGTGGGCTCATTGC-3’
	reverse	5’-CATGAGAATGCCTCCAAACAC-3’
IL-10	forward	5’-GCTCTTACTGACTGGCATGAG-3’
	reverse	5’-CGCAGCTCTAGGAGCATGTG-3’
IL-1β	forward	5’-CCTTCCAGGATGAGGACATGA-3’
	reverse	5’-TGAGTCACAGAGGATGGGCTC-3’
IL-4	forward	5’-CCAAACGTCCTCACAGCAAC-3’
	reverse	5’-AAGCCCGAAAGAGTCTCTGC-3’
IL-6	forward	5’-CACGGCCTTCCCTACTTCAC-3’
	reverse	5’-GGTCTGTTGGGAGTGGTATC-3’
iNOS	forward	5’-GTTCTCAGCCCAACAATACAAGA-3’
	reverse	5’-GTGGACGGGTCGATGTCAC-3’
MFN-2	forward	5’-TGCACCGCCATATAGAGGAAG-3’
	reverse	5’-TCTGCAGTGAACTGGCAATG-3’
TNF-α	forward	5’-CCCTCCTGGCCAACGGCATG-3’
	reverse	5’-TCGGGGCAGCCTTGTCCCTT-3’
MFN1	forward	5’-GCAGACAGCACATGGAGAGA-3’
	reverse	5’-GATCCGATTCCGAGCTTCCG-3’
OPA1	forward	5’-ACCTTGCCAGTTTAGCTCCC-3’
	reverse	5’-TTGGGACCTGCAGTGAAGAA-3’
DRP1	forward	5’-ATGCCAGCAAGTCCACAGAA-3’
	reverse	5’-TGTTCTCGGGCAGACAGTTT-3’
FIS1	forward	5’-CAAAGAGGAACAGCGGGACT-3’
	reverse	5’-ACAGCCCTCGCACATACTTT-3’
SREBP1	forward	5’-GATGTGCGAACTGGACACAG-3’
	reverse	5’-CATAGGGGGCGTCAAACAG-3’
SRBP2	forward	5’-GCAGCAACGGGACCATTCT-3’
	reverse	5’-CCCCATGACTAAGTCCTTCAACT-3’
LDLR	forward	5’-TGACTCAGACGAACAAGGCTG-3’
	reverse	5’-ATCTAGGCAATCTCGGTCTCC-3’
HMGCR	forward	5’-TGTTCACCGGCAACAACAAGA-3’
	reverse	5’-CCGCGTTATCGTCAGGATGA-3’
SRA	forward	5’-TCAGACTGAAGGACTGGGAACA-3’
	reverse	5’-GGAGGCCCTTGAATGAAGGT-3’
SRB1	forward	5’-TGTACTGCCTAACATCTTGGTCC-3’
	reverse	5’-ACTGTGCGGTTCATAAAAGCA-3’
ABCG1	forward	5’-TCACCCAGTTCTGCATCCTCT-3’
	reverse	5’-GCAGATGTGTCAGGACCGAGT-3’
ABCA1	forward	5’-CGTTTCCGGGAAGTGTCCTA-3’
	reverse	5’-GCTAGAGATGACAAGGAGGATGGA-3’
hCETP-THP-1	forward	5’-TCAGTCCATTGACTTCGAGATCG-3’
	reverse	5’-GCATCGGTCCGCACTCTAC-3’
hTNF-α-THP-1	forward	5’-TCAGCAAGGACAGCAGAGG -3’
	reverse	5’-CAGTATGTGAGAGGAAGAGAACC-3’
hMFN-2-THP-1	forward	5’-CACATGGAGCGTTGTACCAG-3’

Gene	Oligonucleotides sequences	
	reverse	5’-TTGAGCACCTCCTTAGCAGAC-3’
hCD36-THP-1	forward	5’-GGCTGTGACCGGAACTGTG-3’
	reverse	5’-AGGTCTCCAACTGGCATTAGAA-3’
hGAPDH-THP-1	forward	5’-CTGGGCTACACTGAGCACC-3’
	reverse	5’-AAGTGGTCGTTGAGGGCAATG-3’
hPGK1-THP-1	forward	5’-GACCTAATGTCCAAAGCTGAGAA-3’
	reverse	5’-CAGCAGGTATGCCAGAAGCC-3’
